# The effect of three different energy drinks on oxygen consumption and perceived exertion during treadmill exercise

**DOI:** 10.1186/1550-2783-12-S1-P1

**Published:** 2015-09-21

**Authors:** Gabriel J Sanders, Willard Peveler, Brady Holmer, Corey A Peacock

**Affiliations:** 1 Northern Kentucky University, Highland Heights, KY, USA; 2 Nova Southeastern University, Fort Lauderdale, FL, USA

## Background

Some energy drink manufacturers claim that their products can increase athletic performance. However, there are no studies to assess the effect of these energy drinks on oxygen consumption (VO_2_) or ratings of perceived exertion (RPE) during exercise. If these energy drinks improve performance, VO_2 _and RPE would likely be reduced during any given exercise intensity.

## Methods

Fifteen (22.1 ± 2.7 years old) participants completed the study. Maximal oxygen consumption (VO_2 _max) was initially measured to establish each participant's exercise for the 70% treadmill exercise protocol after ingesting an energy drink. Following VO_2 _max testing, all participants completed a total of four conditions. Each condition required a participant to ingest an energy drink then rest in a seated position for one hour. Following one hour of rest, participants exercised for a total of 15 minutes on a treadmill at 70% of their VO_2 _max_. _For each condition, participants blindly ingested one of four price-matched beverages (12 oz. placebo (Squirt), 8.4 oz. Red Bull^®^, 16 oz. Monster Energy ^®^, 2 oz. 5-hour ENERGY^®^). Relative VO_2 _(ml.kg^-1.^min^-1^) and RPE (6-20 Borg Scale) were recorded each minute during the treadmill exercise and averaged in five-minute increments and as an average for each 15-minute condition.

## Results

Analysis of variance revealed there was no significant main effect of energy drinks on average VO_2 _(placebo 35.8 ± 2.3 ml.kg^-1.^min^-1^; Red Bull 35.4 ± 2.3 ml.kg^-1.^min^-1^; Monster 35.8 ± 2.2 ml.kg^-1.^min^-1^; 5-hour 36.5 ± 2.4 ml.kg^-1.^min^-1^; p ≥ .482) and RPE (placebo 12.2 ± 0.6; Red Bull 12.6 ± 0.5; Monster 12.0 ± 0.5; 5-hour 11.7 ± 0.5; p ≥ .179) during 15 minutes of treadmill exercise.

## Conclusions

Energy drinks do not appear to improve perceived treadmill exercise performance nor running economy assessed via oxygen consumption at 70% treadmill exercise. Given that no significant reductions were found in VO_2 _and RPE post energy drink consumption, results do not support manufacturers' claims regarding their product's ability to boost performance. Additional research is needed to assess time trial or time to exhaustion sprint and endurance performance. Time trials and time to exhaustion may better assess if these energy drinks can, in fact, improve exercise performance.

